# Lung volumes and lung volume recruitment in ARDS: a comparison between supine and prone position

**DOI:** 10.1186/s13613-018-0371-0

**Published:** 2018-02-14

**Authors:** Hernan Aguirre-Bermeo, Marta Turella, Maddalena Bitondo, Juan Grandjean, Stefano Italiano, Olimpia Festa, Indalecio Morán, Jordi Mancebo

**Affiliations:** Servei de Medicina Intensiva, Hospital de la Santa Creu i Sant Pau, Universitat Autònoma de Barcelona (UAB), Sant Quintí, 89, 08041 Barcelona, Spain

**Keywords:** ARDS, Lung volumes, Lung strain, Prone, PEEP recruitment, Mechanical ventilation

## Abstract

**Background:**

The use of positive end-expiratory pressure (PEEP) and prone position (PP) is common in the management of severe acute respiratory distress syndrome patients (ARDS). We conducted this study to analyze the variation in lung volumes and PEEP-induced lung volume recruitment with the change from supine position (SP) to PP in ARDS patients.

**Methods:**

The investigation was conducted in a multidisciplinary intensive care unit. Patients who met the clinical criteria of the Berlin definition for ARDS were included. The responsible physician set basal PEEP. To avoid hypoxemia, FiO_2_ was increased to 0.8 1 h before starting the protocol. End-expiratory lung volume (EELV) and functional residual capacity (FRC) were measured using the nitrogen washout/washin technique. After the procedures in SP, the patients were turned to PP and 1 h later the same procedures were made in PP.

**Results:**

Twenty-three patients were included in the study, and twenty were analyzed. The change from SP to PP significantly increased FRC (from 965 ± 397 to 1140 ± 490 ml, *p* = 0.008) and EELV (from 1566 ± 476 to 1832 ± 719 ml, *p* = 0.008), but PEEP-induced lung volume recruitment did not significantly change (269 ± 186 ml in SP to 324 ± 188 ml in PP, *p* = 0.263). Dynamic strain at PEEP decreased with the change from SP to PP (0.38 ± 0.14 to 0.33 ± 0.13, *p* = 0.040).

**Conclusions:**

As compared to supine, prone position increases resting lung volumes and decreases dynamic lung strain.

**Electronic supplementary material:**

The online version of this article (10.1186/s13613-018-0371-0) contains supplementary material, which is available to authorized users.

## Background

Acute respiratory distress syndrome (ARDS) is a permeability pulmonary edema, characterized by hypoxemia and a decrease in lung volumes and respiratory system compliance [1, 2]. In patients with ARDS, prone position (PP) produces a more homogeneous distribution of the inspired gas [3] and a better matching between ventilation and perfusion, thereby improving arterial oxygenation [3–5]. Positive end-expiratory pressure (PEEP) and PP have also shown to decrease the percentage of non-aerated and poorly aerated lung tissue and attenuate the regional recruitment–derecruitment phenomena [5–7]. In selected ARDS patients, PP has been proposed to further improve the outcomes [8]. The benefit on survival of PP is not related only to the improvement in gas exchange [9, 10], and the protective effect on ventilator-induced lung injury [3, 9, 11, 12] could also play a role. As compared to supine position (SP), the PP reduces the steep transpulmonary pressure gradient across the vertical axis of the lung, leading to a more homogeneous distribution of pulmonary stress and strain [2, 3, 13].

However, data analyzing the variation in lung volumes with the change from SP to PP in ARDS patients are scarce and conflicting [4, 14–17]. We hypothesized that in ARDS patients, PP increases lung volumes (i.e., functional residual capacity and end-expiratory lung volume) and might decrease lung strain [16, 18]. Because the measurement of functional residual capacity (FRC) requires to be made at zero end-expiratory pressure (ZEEP), our study included a lung derecruitment maneuver from baseline PEEP to zero PEEP [19–21] subsequently followed by the reinstitution of the basal PEEP level. These allowed to analyze the variation in lung volumes and to estimate lung volume recruitment and lung strain in both supine and prone positions in patients with ARDS.

## Methods

The study was performed in the Intensive Care Department at Hospital de la Santa Creu i Sant Pau, Barcelona (Spain). This study was conducted in accordance with the amended Declaration of Helsinki.

### Patients

Patients were considered eligible for the study if they met the Berlin definition criteria for ARDS [22] and had an indication for PP in accordance with our department’s protocol (PaO_2_/FiO_2_ ratio of < 150 mm Hg and FiO_2_ of ≥ 0.6 with PEEP of at least 5 cm H_2_O). We recommend to use protective ventilation with individualized low tidal volume (Vt) and moderate PEEP levels. Essentially, PEEP is titrated according to the gas exchange (Sat O_2_, measured by pulse oxymeter, around 95%) with end-inspiratory plateau airway pressure (Pplat) not higher than 28 cm H_2_O and without hemodynamic instability (mean arterial pressure above 65 mm Hg and no need for fluid replacement). Our detailed ventilatory strategy is included in Additional file 1. Hence, all our patients had been turned in PP before inclusion in the study. To be included, patients had to present an improvement in gas exchange (FiO_2_ ≤ 0.6 and PEEP ≤ 12 cm H_2_O) in SP in order to avoid severe hypoxemia because of the derecruitment (induced by PEEP withdrawal and ventilation at ZEEP) during the measurement of FRC. Exclusion criteria were: age < 18 years, tracheostomy, pregnancy, major trauma, barotrauma (presence of extra-alveolar air during mechanical ventilation as assessed by daily chest X ray) and hemodynamic instability (systolic blood pressure < 80 or > 160 mm Hg, heart rate < 50 bpm or > 130 bpm or changes in ± 20% from baseline).

All patients were under continuous sedation and analgesia with intravenous perfusion of midazolam and/or propofol and opioids. During the study period, all patients received neuromuscular blocking agents.

### Protocol

The following data were collected: age, height, simplified acute physiology score III at admission, ARDS etiology, days of mechanical ventilation, intensive care unit outcomes, respiratory rate, Vt, PEEP, peak airway pressure, Pplat and arterial blood gases. Respiratory variables were recorded directly from the ventilator.

All patients were ventilated in volume control ventilation using the same ventilator model (Engström Carestation ICU ventilator, General Electric, Madison, WI, USA).

To avoid hypoxemia, defined as oxygen saturation ≤ 88% measured through pulse oximetry, we increased the FiO_2_ to 0.8 1 h before starting the protocol.

### Measurements

**Fig. 1:**
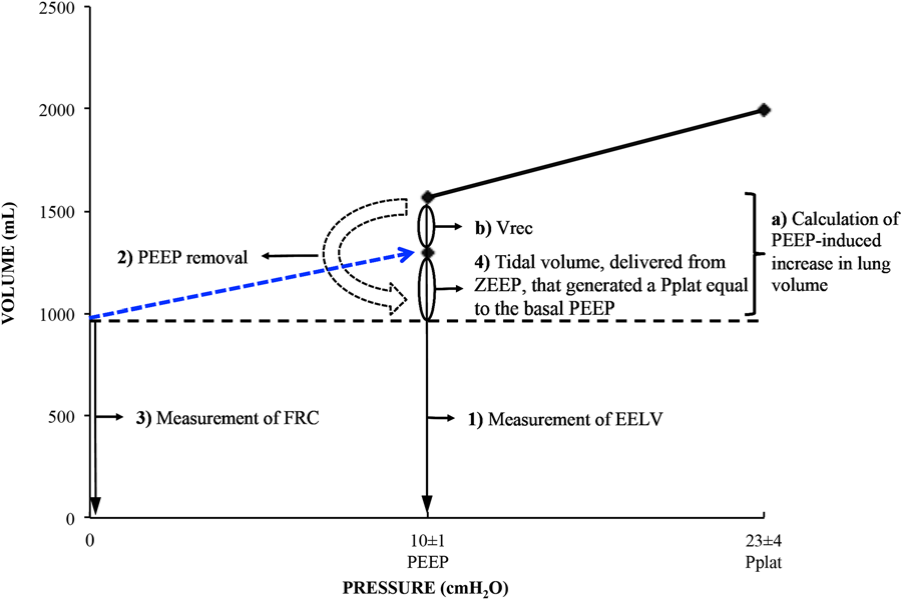
Lung volumes, measurements and calculations made in the study. The same procedures were carried out in supine and prone positions as follows: (1) measurement of end-expiratory lung volume (EELV): EELV is defined as the resting end-expiratory lung volume at PEEP. (2) Removal of PEEP and continuation of mechanical ventilation at zero end-expiratory pressure (ZEEP). (3) Measurement of functional residual capacity (FRC): FRC is defined as the resting lung volume at ZEEP. (4) Measurement of the tidal volume, delivered from ZEEP, that generated a Pplat equal to the basal PEEP. The same calculations were carried out in supine and prone positions as follows: (a) calculation of PEEP-induced increase in lung volume = EELV minus FRC. (b) Calculation of PEEP-induced lung volume recruitment (Vrec) = PEEP-induced increase in lung volume minus the Vt, delivered from ZEEP, that generated a Pplat equal to the basal PEEP. Blue line represents the compliance at ZEEP

Baseline ventilatory and hemodynamic parameters were collected before the protocol to measure lung volumes. The same procedures were carried out in SP and PP and are outlined below (see also Fig. 1):

1. Measurement of end-expiratory lung volume (EELV): EELV is the resting end-expiratory lung volume measured at baseline PEEP.
2. Removal of PEEP and continuation of mechanical ventilation at ZEEP. This derecruitment maneuver is mandatory to conduct the following step 3, and it is the reason to increase the FiO_2_ to 0.8 immediately before starting the protocol (i.e., to avoid hypoxemia).
3. Measurement of functional residual capacity (FRC): FRC is the resting lung volume measured at ZEEP.
4. Measurement of the Vt, delivered from ZEEP, that generated a Pplat equal to the basal PEEP. This step (see Fig. 1) is mandatory to allow a proper estimation of the PEEP-induced lung volume recruitment [19, 20, 23–25].

Once step 4 was completed, the same PEEP that was used at baseline was resumed.

Measurements at ZEEP (FRC and Vt delivered from ZEEP, that generated a Pplat equal to the basal PEEP) included a lung derecruitment maneuver (PEEP removal) that can produce hypoxemia. For the purpose of our investigation, we defined hypoxemia as oxygen saturation ≤ 88% measured through pulse oximetry.

The safety limits and contraindications to remove PEEP were:

1. PEEP removal was contraindicated if FiO_2_ > 0.6 and PEEP > 12 cm H_2_O.
2. We increased the FiO_2_ to 0.8 1 h before starting the protocol in order to avoid hypoxemia during PEEP removal.
3. If a patient presented with hypoxemia at any time during the protocol (saturation ≤ 88% measured through pulse oximetry), the measurements were aborted and the patient was excluded.

Lung volumes (EELV and FRC) were measured twice using the nitrogen washout/washin technique available in Engström Carestation ICU ventilator as previously described [24, 26]. Washout/washin technique is a multiple breath maneuver that with a modification of 0.1 in FiO_2_ calculates the residual nitrogen in the lung (assuming there is not exchange of nitrogen) by continuous measurements of oxygen and carbon dioxide. The ventilator was carefully calibrated before the measurements according to the manufacturer’s specifications. We obtained four values for each lung volume. The mean of the four values was used. As previously suggested [27], patients were excluded if the differences between the four values were more than 20% (cutoff determined by the manufacturer).

After the procedures in SP, the patients were turned to PP and 1 h later the same procedures (from 1 to 4 above) were made in PP. This time span was based in previous data showing that after 1 h in PP gas exchange is stable in the majority of patients [28, 29]. If a patient presented with hypoxemia (oxygen saturation ≤ 88%) at any time during the protocol, the measurements were aborted and the patient was excluded.

The normal reference values for FRC (liters) in the SP were calculated according to the equation described by Ibáñez and Raurich [30], as follows: 5.48 × height—7.05 for men and 1.39 × height—0.424 for women; height units are in meters. Compliance (ml/cm H_2_O) was calculated as Vt/(Pplat minus total PEEP), being total PEEP the sum of PEEP plus intrinsic PEEP. Predicted body weight was calculated as follows: 50 + 0.91(height—152.4) for men and 45.5 + 0.91(height—152.4) for women; height units are in centimeters. Driving airway pressure was calculated as the difference between Pplat and total PEEP [31].

### Calculation of lung volumes and strain

- The PEEP-induced increase in lung volume was calculated as EELV minus FRC (see Fig. 1).
- PEEP-induced lung volume recruitment (Vrec) was calculated as PEEP-induced increase in lung volume minus the Vt, delivered from ZEEP, that generated a Pplat equal to the basal PEEP (see Fig. 1).
- Strain was calculated as previously described [24, 32, 33]:
  1. Dynamic strain at ZEEP = Vt/FRC.
  2. Dynamic strain at PEEP = Vt/(FRC + Vrec).
  3. Static strain at PEEP = (EELV − FRC)/(FRC + Vrec).
  4. Global strain at PEEP = (static strain at PEEP + dynamic strain at PEEP) = (EELV − FRC + Vt)/(FRC + Vrec).

### Statistical analysis

Data are expressed as mean ± SD. We used Wilcoxon test to compare variables between supine and prone positions and *U* the Mann–Whiney test to compare early and non-early ARDS patients. A *p* value < 0.05 was considered statistically significant. The SPSS^®^ Statistics (version 20.0, Chicago, IL, USA) statistical software was used for statistical analysis.

## Results

The study was conducted from July 2010 to December 2013. Twenty-three patients were included in the study, and twenty were analyzed. One patient was excluded because of hypoxemia during the FRC measurement, and two were excluded because of a technical problem. (The differences between FRC measurements were > 20%.)

Table 1 summarizes the patients’ main characteristics at baseline. The mean age of patients was 58 ± 18 years. The main causes of ARDS were pneumonia (*n* = 11) and septic shock (*n* = 4). The study was performed 4 ± 3 days after starting mechanical ventilation. At baseline, mean Vt was 6.9 ± 1.4 ml/kg of predicted body weight and mean PEEP was 10 ± 1 cm H_2_O.

**Table 1:** Patients’ characteristics at study entry (with FiO_2_ 0.8)

Patient	Age (years)	Days on MV before study	SAPS III	Vt (ml/kg PBW)	RR (rpm)	PEEP (cm H_2_O)	Pplat (cm H_2_O)	Δ Paw (cm H_2_O)	PaO_2_/FiO_2_ (mm Hg)	PaCO_2_ (mm Hg)	Cause of ARDS	Outcome
1	43	6	65	7.4	24	8	28	20	255	40	Pneumonia	S
2	66	1	52	6.1	22	10	20	10	254	60	Pneumonia	S
3	77	5	91	6.7	20	10	22	12	165	44	Pneumonia	S
4	68	4	69	8.4	24	12	21	9	255	44	Pneumonia	S
5	75	4	65	7.8	22	10	18	8	115	38	Pneumonia	S
6	65	7	94	9.2	30	10	21	11	240	53	Pneumonia	S
7	55	2	67	6.8	20	10	22	12	229	35	Peritonitis	S
8	43	2	77	8.1	20	8	26	18	151	48	Peritonitis	D
9	78	3	100	6.3	27	10	29	19	188	41	Peritonitis	D
10	74	2	82	11.0	25	10	28	18	198	43	Pneumonia	D
11	81	4	89	6.7	24	10	20	10	230	34	Septic shock	S
12	30	4	83	6.8	21	10	23	13	265	41	Septic shock	D
13	58	5	71	5.0	30	10	20	10	173	43	Pneumonia	S
14	69	1	101	5.9	28	10	28	18	300	44	Septic shock	D
15	50	14	95	6.0	30	10	25	15	206	42	Septic shock	D
16	55	3	68	7.0	20	8	19	11	129	37	Thoracic Trauma	S
17	30	4	64	6.2	24	12	22	10	104	41	Pneumonia	S
18	37	3	76	5.0	30	12	22	10	299	29	Pneumonia	D
19	80	2	82	6.1	17	12	19	7	230	35	Pneumonia	S
20	31	5	65	6.7	26	12	27	15	218	24	Pancreatitis	D
Mean ± SD	58 ± 18	4 ± 3	78 ± 14	6.9 ± 1.4	24 ± 4	10 ± 1	23 ± 4	13 ± 4	41 ± 8	41 ± 8		

*ARDS* acute respiratory distress syndrome, *D* died, *PBW* predicted body weight, *MV* mechanical ventilation, *PEEP* positive end-expiratory pressure, *Pplat* end-inspiratory plateau airway pressure, *RR* respiratory rate, *S* survived, *SAPS III* simplified acute physiology score III, *Vt* tidal volume, *Δ Paw* driving airway pressure

After assuming the PP, the PaO_2_/FiO_2_ ratio increased significantly, from 210 ± 57 mm Hg in supine to 281 ± 109 mm Hg in prone (*p* = 0.008) (Table 2).

**Table 2:** Main characteristics of all patients in each position

Variable	Supine *n* = 20	Prone *n* = 20	*p*
PaO_2_/FiO_2_ (mm Hg)	210 ± 57	281 ± 109	0.021
PaCO_2_ (mm Hg)	41 ± 8	42 ± 9	0.400
Peak airway pressure (cm H_2_O)	41 ± 7	41 ± 6	0.284
Pplat (cm H_2_O)	23 ± 4	23 ± 4	0.446
Compliance (ml/cm H_2_O)	36 ± 11	37 ± 10	0.594
Δ Paw (cm H_2_O)	13 ± 4	12 ± 4	0.446
FRC (ml)	965 ± 397	1140 ± 490	0.021
EELV (ml)	1566 ± 476	1832 ± 719	0.009
Vt delivered from ZEEP, that generated a Pplat equal to basal PEEP [ml (*n* = 16)]	333 ± 105	360 ± 127	0.073
Vrec [ml (*n* = 16)]	269 ± 186	324 ± 188	0.501
Dynamic strain at ZEEP	0.52 ± 0.23	0.44 ± 0.18	0.040
Dynamic strain at PEEP (*n* = 16)	0.38 ± 0.14	0.33 ± 0.13	0.020
Static strain at PEEP (*n* = 16)	0.51 ± 0.16	0.48 ± 0.13	0.438
Global strain at PEEP (*n* = 16)	0.89 ± 0.24	0.81 ± 0.18	0.121

Data are presented as mean ± SD. Dynamic strain at ZEEP = Vt/FRC; dynamic strain at PEEP = Vt/(FRC + Vrec); static strain at PEEP = (EELV − FRC)/(FRC + Vrec); global strain at PEEP = (EELV − FRC + Vt)/(FRC + Vrec)

*EELV* end-expiratory lung volume, *FRC* functional residual capacity, *PEEP* positive end-expiratory pressure, *Pplat* end-inspiratory plateau airway pressure, *Vrec* PEEP-induced lung volume recruitment, *Vt* tidal volume, *Δ Paw* driving airway pressure

**Fig. 2:**
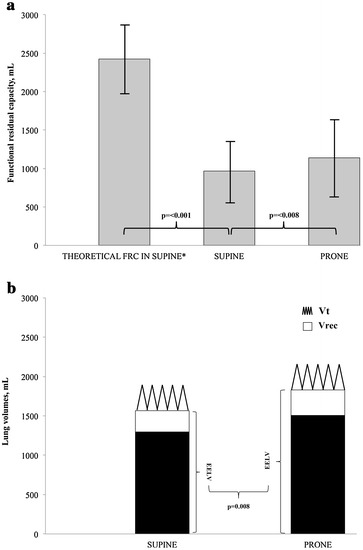
Variation of lung volumes with the change of position. **a** Comparison of different values of functional residual capacity. **b** Comparison of EELV and Vrec in supine position and prone position. EELV, end-expiratory lung volume; Vrec, PEEP-induced lung volume recruitment; Vt, tidal volume. Data are presented in mean (ml) and SD. *According to the equation described by Ibañez and Raurich [30]

**Fig. 3:**
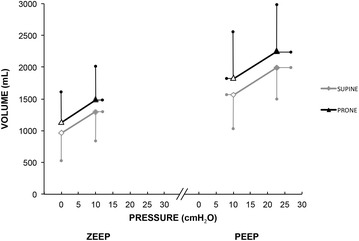
Variation in lung volumes in supine and prone positions. Clear triangles and clear rhombus are the resting lung volumes at ZEEP and at PEEP. Dark triangles and dark rhombus represent end-inspiratory lung volumes and end-inspiratory lung pressure (Pplat) at ZEEP and at PEEP. PEEP, positive end-expiratory pressure; ZEEP, zero end-expiratory pressure. Data are shown as mean and SD

The mean FRC in SP was significantly lower than its reference value in healthy normal subjects (965 ± 397 vs. 2424 ± 459 ml, *p* ≤ 0.001). The change from SP to PP significantly increased both FRC (from 965 ± 397 to 1140 ± 490 ml, *p* = 0.008) and EELV (from 1566 ± 476 to 1832 ± 719 ml, *p* = 0.008) (Figs. 2, 3).

We did not calculate Vrec and derived parameters in four patients because the tidal volume delivered from ZEEP, that generated a Pplat equal to the basal PEEP, was not measured in accordance to the protocol. Vrec (n = 16) did not significantly vary with the change of position (269 ± 186 ml in SP to 324 ± 188 ml in PP, *p* = 0.263) (Fig. 2).

**Fig. 4:**
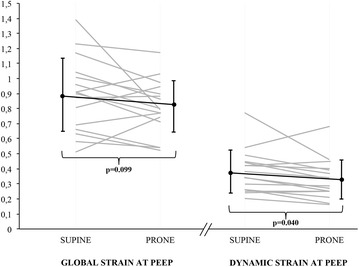
Variation of individual values of global and dynamic strain at PEEP with the change of position. Dark lines represent mean and SD. PEEP, positive end-expiratory pressure

We found a significant decrease in the dynamic strain at PEEP with the change from SP to PP from 0.38 ± 0.14 to 0.33 ± 0.13 (*p* = 0.040) (Fig. 4). The dynamic strain at ZEEP also decreased, from 0.52 ± 0.23 in SP to 0.44 ± 0.18 in PP (*p* = 0.047). The remaining variables did not change significantly between supine and prone positions (Table 2) (Additional file 2: Table S1).

In the whole population, the driving pressure in the non-survivor group (*n* = 8) was significantly higher than in the survivor group (*n* = 12) in both SP (16 ± 3 cm H_2_O vs. 11 ± 3 cm H_2_O, respectively, *p* = 0.003) and in PP (15 ± 3 cm H_2_O vs. 11 ± 3 cm H_2_O, respectively, *p* = 0.005). Additional data are also shown (Additional file 2: Table S2).

## Discussion

The main findings in this study were that: (1) Prone position significantly increased lung volumes; (2) dynamic strain decreased significantly in prone position compared to supine position; and (3) the change of position from supine to prone did not modify the calculated PEEP-induced lung volume recruitment.

### Prone position, oxygenation and lung volumes

In ARDS patients, lung volumes at ZEEP (FRC) and at PEEP (EELV) are typically decreased [18]. Two previous studies have shown that PP significantly increases FRC in ARDS patients [15, 16]. Nevertheless, data about the changes in EELV with the change from SP to PP in ARDS patients are not consistent. Four previous studies have shown that PP increases EELV in ARDS patients as compared to SP [14–17], but another study [4] found that the change of EELV from SP to PP was not significant. These contradictory findings might be explained by differences in lung recruitability, distribution and extension of lung volume alterations, differences in chest wall compliance, the influence of abdominal weight and heart compression, the inclination from the horizontal plane and the use or not of ventral supports [3, 9, 34, 35].

In the present study, we found a 40% decrease in FRC as compared to its reference value in SP, confirming previous results [18]. We also observed that the FRC and EELV increased significantly with the change of position (18% in FRC and 17% in EELV). Santini et al. [7] performed a study in animals with normal lungs, and they found a significant increase in FRC with the change from SP to PP. The increase in resting lung volume was mainly related to a redistribution of aeration: a minor decrease in non-aerated lung tissue (3%), a major decrease in poorly aerated tissue (17%) and a major increase (20%) in well-aerated tissue. Since recruitment, as precisely measured by thoracic CT scan, refers to tissue recruitment (i.e., amount of non-inflated tissue that reinflates at a higher pressure), the decrease in poorly aerated tissue and the increase in well-aerated tissue (which contribute to the end-expiratory lung volume increase induced by PEEP) are thus considered as better gas distribution within the lung and not recruitment per se [36].

### Prone position and strain

During passive mechanical ventilation, the force applied by the ventilator generates an internal tension in the fibers of the lung skeleton, called “stress,” and the elongation of these fibers from their resting position is called “strain” [2]. High values of dynamic lung strain (lung deformation caused by Vt) and static lung strain (lung deformation caused by PEEP) are associated with ventilator-induced lung injury [32, 37].

In an animal model, Protti et al. [33] showed that for the same global strain, a large static strain is less harmful than a large dynamic strain. On the same vein, González-López et al. [38] found that increased strain was associated with a proinflammatory lung response in patients with acute lung injury. Moreover, Bellani et al. [39] found in patients with acute lung injury that the intensity of metabolic activity (a surrogate of inflammation) detected by positron emission tomography was correlated with regional strain. Consequently, the significant decrease in dynamic strain in PP as compared to SP could be another mechanism of protection of PP against ventilator-induced lung injury. Therefore, the measurement of lung volumes at bedside may be an important tool to deliver a more physiologically based ventilation and encourage physicians to increase the use of PP in moderately to severe ARDS patients [40].

### Prone position and PEEP-induced lung volume recruitment

It is still unclear whether the PEEP-induced alveolar recruitment varies with the change from SP to PP. In an experimental study in animals with lung injury, Richard et al. [5] analyzed the variation of alveolar recruitment at PEEP 10 cm H_2_O in SP and PP by means of the positron emission tomography technique. They found that in PP, PEEP-induced alveolar recruitment was not higher than in SP. Interestingly, in this study, the authors observed a redistribution of densities in PP (recruitment in dorsal regions with derecruitment in ventral regions). Cornejo et al. [6] performed another study in ARDS patients to determine the effects of PEEP and PP on alveolar recruitment. Using the CT scan technique, they found that increasing PEEP from 5 cm H_2_O to 15 cm H_2_O significantly increased alveolar recruitment. However, the percentage of recruitment was similar in both positions (36% in SP and 33% in PP). Using a different methodology, the data from our study are consistent with these findings, indicating that the effects of PEEP on lung volume recruitment are similar in both positions (around 17% of EELV).

A previous study by Grasso et al. [41] found that alveolar recruitment was higher in the early phase (1 ± 0.3 days of mechanical ventilation) than in the late phase of ARDS, but a subsequent study by Gattinoni et al. [42] did not find the same results. In the study of Gattinoni et al. [42], they found that the number of days of mechanical ventilation before the study was similar in patients with a lower percentage of potentially recruitable lung and those with a higher percentage (5 ± 6 vs. 6 ± 6 days, respectively, *p* = 0.50). In our study when we classified ARDS patients in the early phase (< 72 h) and in late phase (> 72 h) (Additional file 2: Table S2), we observed results similar to those of Gattinoni et al. [42]: no statistical differences in lung volume recruitment between the early and late phase group were detected. In our study, however, in the early phase of ARDS, lung volumes increased and strain decreased with the change from SP to PP, whereas in late phase ARDS we did not observe these findings (Additional file 2: Table S2). These differences could be related to the presence of some degree of hydrostatic pulmonary edema in the early phase of ARDS, and to the presence of fibrosis in the non-early phase of ARDS that predisposes to non-responsiveness to PP in terms of increasing in lung volumes and decreasing strain [43]. Our findings thus suggest that the survival benefit may, in part, be related to the early application of PP as it increases resting lung volumes and decreases lung strain compared to SP. It is also tempting to speculate that the lack of differences in Vrec between supine and prone, and the increase in overall lung volume in prone as compared to supine, can be explained by a decrease of poorly ventilated areas and an increase of well ventilated areas, which in turn might help to decrease lung inhomogeneity. It has been shown that the extent of lung inhomogeneities (as quantified by the amount of poorly ventilated tissue) is associated with worse outcomes in ARDS patients, possibly due to a mechanism of “stress raisers” [44].

### Limitations

Like many physiological studies [4, 6, 14–17, 34], our study has a relatively low number of patients. Another limitation is that the measurement of FRC could be subject to the tolerance to PEEP removal and the FiO_2_ used. However, when the study was performed, all the patients met the criteria for mild–moderate ARDS according to the Berlin definition [22]. We did not perform a multi-slice spiral lung computed tomography to measure the quantitative changes in alveolar aeration induced by PEEP and PP. Other measurements of lung mechanics (i.e., esophageal pressure and derived variables) and lung biomarkers could help to further explain the effects of PEEP and positioning in ARDS patients, but we did not do these because of lack of adequate equipment at the time of the study. Finally, to confirm the changes, it might have been useful to return the patients from PP to SP and to repeat the same procedures and measurements; this was not done, however, because most patients remained in the PP as per clinical decision after the study had been completed.

## Conclusions

As compared to supine, prone position increases resting lung volumes without significantly changing the recruited volume kept by PEEP. Moreover, the change of position from supine to prone decreases dynamic lung strain. These findings help to better understand the beneficial effects of prone position in ARDS patients.

## Additional files

**Additional file 1.** The institutional protocol.

**Additional file 2.** Additional patient per patient physiological data in supine and prone position (Table S1 and Table S2).

## Abbreviations

ARDS: acute respiratory distress syndrome
EELV: end-expiratory lung volume
FRC: functional residual capacity
PEEP: positive end-expiratory pressure
PP: prone position
Pplat: end-inspiratory plateau airway pressure
SP: supine position
Vrec: PEEP-induced lung volume recruitment
Vt: tidal volume
ZEEP: zero end-expiratory pressure
