# Supplementary material for: Lung volumes and lung volume recruitment in ARDS: a comparison between supine and prone position
Source: Ann Intensive Care. 2018 Feb 14;8:25. doi: 10.1186/s13613-018-0371-0 (PMC5812959; doi:10.1186/s13613-018-0371-0)
Supplement: Supplementary file 1 — Additional file 1. The institutional protocol. [file 13613_2018_371_MOESM1_ESM.docx]

Institutional Protocol

Our institutional protocol is based on previous data and experience[1-3] and is detailed below.

Mechanical ventilation settings in ARDS patients

The volume assist-control mode with constant inspiratory flow is used. We recommend to use protective ventilation with individualized low tidal volume (Vt) and moderate PEEP levels. Essentially, PEEP is titrated according to the gas exchange (Sat O2, measured by pulse oxymeter, around 95%) with end-inspiratory plateau airway pressure (Pplat) not higher than 28 cm H2O and without hemodynamic instability (mean arterial pressure above 65 mmHg and no need for fluid replacement), and an inspiratory to expiratory ratio ≤ 1:2 have to be set immediately after ARDS diagnosis.

Prone Position

If patient have a PaO_2_/FiO_2_ ratio of <150 mm Hg and FiO_2_ of ≥0.6 with PEEP of at least 5 cm H_2_O the physician must to consider prone position. The patients have to be maintained in PP for at least 16 consecutive hours. The prone sessions could be stopped when PaO_2_/FIO_2_ ratio are ≥ 150 mmHg with PEEP ≤ 10 cm H_2_O and FiO_2_ ≤0.6 measured in supine position and after one our of the last prone position phase.

Five people were usually involved and the process typically took five to ten minutes. One person has to be dedicated to the management of the head of the patient, the endotracheal tube and the ventilator lines. This person at the head of the bed had to coordinate the steps of the procedure.

After the turn, the patients’ arms were placed alongside their bodies. Their heads and necks were moved from lateral to midline to contralateral positions at varying intervals. Special care was taken to protect the eyes. A slight reverse Trendelenburg position is used to minimize facial edema.

Neuromuscular blocking agents are used early and have to be stopped at the discretion of the attending physician from 48 hours of ARDS treatment.

In supine and prone position the inclination was 30° from the horizontal.

Weaning

When the prone sessions were stopped the physician could considered to start weaning phase. First we made a gradually decrease of PEEP (steps of 2 cm H_2_O) up to 8 cm H_2_O (keeping arterial oxygen saturation between 93 and 98%, estimated by a pulse oximeter). Then FiO_2_ was gradually decreased to 0.45 to keep the same arterial oxygen saturation. At this time, the use of pressure support ventilation could be considered. The initial level of pressure support was titrated to achieve a respiratory rate of 25-30 breaths/minute and was decreased twice per day (2-4 cm H_2_O each time) as clinically tolerated. Lastly, PEEP was decreased to 5 cm H2O. At this moment, extubation had to be considered if patients are capable of resuming spontaneous breathing. Spontaneous breathing trial was conducted either with pressure support ventilation (7cm H_2_O of pressure support at ZEEP) or T-piece trials in 30 – 120 minutes. Spontaneous breathing trials were performed once or twice per day.

References:

1. Rialp G, Betbesé AJ, Pérez-Márquez M, Mancebo J. Short-term effects of inhaled nitric oxide and prone position in pulmonary and extrapulmonary acute respiratory distress syndrome. Am J Respir Crit Care Med. 2001;164:243-9.

2. Mancebo J, Fernández R, Blanch L, Rialp G, Gordo F, Ferrer M, Rodriguez F, Garro P, Ricart P, Vallverdú I, Gich I, Castaño J, Saura P, Domínguez G, Bonet A, Albert RK. A multicenter trial of prolonged prone ventilation in severe acute respiratory distress syndrome. Am J Respir Crit Care Med. 2006;173:1233-9.

3. Guérin C, Reignier J, Richard JC, Beuret P, Gacouin A, Boulain T, Mercier E, Badet M, Mercat A, Baudin O, Clavel M, Chatellier D, Jaber S, Rosselli S, Mancebo J, Sirodot M, Hilbert G, Bengler C, Richecoeur J, Gainnier M, Bayle F, Bourdin G, Leray V, Girard R, Baboi L, Ayzac L. Prone positioning in severe acute respiratory distress syndrome. N Engl J Med. 2013;368:2159-68.
