# Supplementary material for: Lung volumes and lung volume recruitment in ARDS: a comparison between supine and prone position
Source: Ann Intensive Care. 2018 Feb 14;8:25. doi: 10.1186/s13613-018-0371-0 (PMC5812959; doi:10.1186/s13613-018-0371-0)
Supplement: Supplementary file 2 — Additional file 2. Additional patient per patient physiological data in supine and prone position (Table S1 and Table S2). [file 13613_2018_371_MOESM2_ESM.doc]

| **Table S1.** Patients’ respiratory characteristics at each position. | | | | | | | | | | | | | | | | |
| --- | --- | --- | --- | --- | --- | --- | --- | --- | --- | --- | --- | --- | --- | --- | --- | --- |
|  | Supine Position | | | | | | | | Prone Position | | | | | | | |
| Patient | Vt at ZEEP (ml) | Pplat at ZEEP (cmH_2_O) | Crs at ZEEP (ml/cm H_2_O) | ∆ Paw at ZEEP (cm H_2_O) | Vt at PEEP (ml) | Pplat at PEEP (cmH_2_O) | Crs at PEEP (ml/cm H_2_O) | ∆ Paw at PEEP (cm H_2_O) | Vt at ZEEP (ml) | Pplat at ZEEP (cmH_2_O) | Crs at ZEEP (ml/cm H_2_O) | ∆ Paw at ZEEP (cm H_2_O) | Vt at PEEP (ml) | Pplat at PEEP (cmH_2_O) | Crs at PEEP (ml/cm H_2_O) | ∆ Paw at PEEP (cm H_2_O) |
| 1 |  |  |  |  | 500 | 28 | 25 | 20 |  |  |  |  | 500 | 26 | 28 | 18 |
| 2 |  |  |  |  | 425 | 20 | 43 | 10 |  |  |  |  | 425 | 20 | 43 | 10 |
| 3 |  |  |  |  | 350 | 22 | 29 | 12 |  |  |  |  | 350 | 21 | 32 | 11 |
| 4 |  |  |  |  | 475 | 21 | 53 | 9 |  |  |  |  | 475 | 24 | 40 | 12 |
| 5 | 375 | 10 | 38 | 10 | 425 | 18 | 53 | 8 | 400 | 10 | 40 | 10 | 425 | 18 | 53 | 8 |
| 6 | 280 | 10 | 28 | 10 | 450 | 21 | 41 | 11 | 300 | 10 | 30 | 10 | 450 | 22 | 38 | 12 |
| 7 | 325 | 10 | 33 | 10 | 450 | 22 | 38 | 12 | 450 | 10 | 45 | 10 | 450 | 20 | 45 | 10 |
| 8 | 160 | 8 | 20 | 8 | 475 | 26 | 26 | 18 | 200 | 8 | 25 | 8 | 475 | 21 | 37 | 13 |
| 9 | 185 | 10 | 19 | 10 | 350 | 29 | 18 | 19 | 200 | 10 | 20 | 10 | 350 | 30 | 18 | 20 |
| 10 | 300 | 10 | 30 | 10 | 425 | 28 | 24 | 18 | 325 | 10 | 33 | 10 | 425 | 25 | 28 | 15 |
| 11 | 525 | 10 | 53 | 10 | 475 | 20 | 48 | 10 | 525 | 10 | 53 | 10 | 475 | 20 | 48 | 10 |
| 12 | 230 | 10 | 23 | 10 | 375 | 23 | 29 | 13 | 220 | 10 | 22 | 10 | 375 | 23 | 29 | 13 |
| 13 | 350 | 10 | 35 | 10 | 375 | 20 | 38 | 10 | 475 | 10 | 48 | 10 | 375 | 20 | 38 | 10 |
| 14 | 325 | 10 | 33 | 10 | 375 | 28 | 21 | 18 | 350 | 10 | 35 | 10 | 375 | 29 | 20 | 19 |
| 15 | 350 | 10 | 35 | 10 | 475 | 25 | 32 | 15 | 370 | 10 | 37 | 10 | 475 | 22 | 40 | 12 |
| 16 | 350 | 8 | 44 | 8 | 450 | 19 | 41 | 11 | 250 | 8 | 31 | 8 | 450 | 20 | 38 | 12 |
| 17 | 375 | 12 | 31 | 12 | 400 | 22 | 40 | 10 | 350 | 12 | 29 | 12 | 400 | 20 | 50 | 8 |
| 18 | 255 | 12 | 21 | 12 | 350 | 22 | 35 | 10 | 375 | 12 | 31 | 12 | 350 | 23 | 32 | 11 |
| 19 | 550 | 12 | 46 | 12 | 400 | 19 | 57 | 7 | 675 | 12 | 56 | 12 | 400 | 19 | 57 | 7 |
| 20 | 400 | 12 | 33 | 12 | 500 | 27 | 33 | 15 | 300 | 12 | 25 | 12 | 500 | 30 | 28 | 18 |
| Mean ± SD | 333 ± 105 | 10 ± 1 | 33 ± 10 | 10 ± 1 | 425 ± 51 | 23 ± 4 | 36 ± 11 | 13 ± 4 | 360 ± 127 | 10 ± 1 | 35 ± 11 | 10 ± 1 | 425 ± 51 | 23 ± 4 | 37 ± 10 | 12 ± 4 |
| Data are presented as mean ± standard deviation.  Abbreviations: Crs, Compliance; PEEP, positive end-expiratory pressure; Pplat, end-inspiratory plateau airway pressure; Vt, Tidal volume; Vt at ZEEP, Vt delivered from ZEEP, that generated a Pplat equal to basal PEEP; ZEEP, Zero end-expiratory pressure; ∆ Paw, Driving airway pressure. | | | | | | | | | | | | | | | | |

| | **Table S2.** Main characteristics of patients in each position according to number of days of mechanical ventilation before the study. | | | | | | | | | | --- | --- | --- | --- | --- | --- | --- | --- | --- | | Variable |  | Early | | |  | Late | | | |  | Supine n=9 | Prone n=9 | P |  | Supine n=11 | Prone n=11 | P | | Vt, ml/Kg of PBW (n=20) |  | 6.9 ± 1.7 | 6.9 ± 1.7 |  |  | 7.0 ± 1.2 | 7.0 ± 1.2 |  | | PEEP, cm H_2_O (n=20) |  | 10 ± 1 | 10 ± 1 |  |  | 10 ± 1 | 10 ± 1 |  | | PaO_2_/FiO_2_, mm Hg |  | 220 ± 60 | 302 ± 128 | 0.093 |  | 202 ± 56 | 264 ± 94 | 0.075 | | PaCO_2_, mm Hg |  | 41 ± 9 | 42 ± 10 | 0.999 |  | 40 ± 7 | 42 ± 9 | 0.182 | | Peak airway pressure, cm H_2_O |  | 39 ± 5 | 42 ± 5 | 0.064 |  | 42 ± 9 | 41 ± 7 | 0.905 | | Pplat, cm H_2_O |  | 24 ± 4 | 23 ± 4 | 0.491 |  | 22 ± 3 | 22 ± 3 | 0.932 | | Compliance, ml/cm H_2_O |  | 34 ± 12 | 35 ± 12 | 0.499 |  | 38 ± 10 | 38 ± 9 | 0.999 | | ∆ Paw, cm H_2_O |  | 14 ± 5 | 13 ± 4 | 0.491 |  | 12 ± 3 | 12 ± 3 | 0.932 | | FRC, ml |  | 851 ± 332 | 1085 ± 354 | 0.011 |  | 1058 ± 436 | 1185 ± 593 | 0.424 | | EELV, ml |  | 1388 ± 435 | 1772 ± 557 | 0.011 |  | 1711 ± 477 | 1880 ± 853 | 0.248 | | Vt delivered from ZEEP, that generated a Pplat equal to basal PEEP, ml (n=16) |  | 306 ± 120 (n=8) | 353 ± 157 (n=8) | 0.068 |  | 361 ± 87 (n=8) | 368 ± 99 (n=8) | 0.671 | | Vrec, ml (n=16) |  | 237 ± 132 (n=8) | 275 ± 136 (n=8) | 0.674 |  | 302 ± 233 (n=8) | 374 ± 228 (n=8) | 0.575 | | Dynamic strain at ZEEP |  | 0.57 ± 0.28 | 0.41 ± 0.12 | 0.011 |  | 0.47 ± 0.17 | 0.46 ± 0.21 | 0.722 | | Dynamic strain at PEEP, (n=16) |  | 0.43 ± 0.16 (n=8) | 0.33 ± 0.08 (n=8) | 0.012 |  | 0.33 ± 0.12 (n=8) | 0.33 ± 0.17 (n=8) | 0.674 | | Static strain at PEEP, (n=16) |  | 0.53 ± 0.13 (n=8) | 0.49 ± 0.16 (n=8) | 0.484 |  | 0.49 ± 0.19 (n=8) | 0.47 ± 0.10 (n=8) | 0.673 | | Global strain at PEEP, (n=16) |  | 0.96 ± 0.23 (n=8) | 0.82 ± 0.16 (n=8) | 0.093 |  | 0.83 ± 0.25 (n=8) | 0.80 ± 0.21 (n=8) | 0.484 | | Data are presented as mean ± standard deviation. Abbreviations: EELV, end-expiratory lung volume; FRC, functional residual capacity; PBW, Predicted body weight; PEEP, Positive end-expiratory pressure; Pplat, end-inspiratory plateau airway pressure; Vrec, PEEP-induced lung volume recruitment; Vd/Vt, dead space; Vt, tidal volume; ∆ Paw, Driving airway pressure (VT / Crs). Dynamic strain at ZEEP = Vt / FRC; Dynamic strain at PEEP = Vt / (FRC + Vrec); Static strain at PEEP = (EELV - FRC) / (FRC + Vrec); Global strain at PEEP = (EELV - FRC + Vt) / (FRC + Vrec). | | | | | | | | | |
| --- | --- | --- | --- | --- | --- | --- | --- | --- | --- | --- | --- | --- | --- | --- | --- | --- | --- | --- | --- | --- | --- | --- | --- | --- | --- | --- | --- | --- | --- | --- | --- | --- | --- | --- | --- | --- | --- | --- | --- | --- | --- | --- | --- | --- | --- | --- | --- | --- | --- | --- | --- | --- | --- | --- | --- | --- | --- | --- | --- | --- | --- | --- | --- | --- | --- | --- | --- | --- | --- | --- | --- | --- | --- | --- | --- | --- | --- | --- | --- | --- | --- | --- | --- | --- | --- | --- | --- | --- | --- | --- | --- | --- | --- | --- | --- | --- | --- | --- | --- | --- | --- | --- | --- | --- | --- | --- | --- | --- | --- | --- | --- | --- | --- | --- | --- | --- | --- | --- | --- | --- | --- | --- | --- | --- | --- | --- | --- | --- | --- | --- | --- | --- | --- | --- | --- | --- | --- | --- | --- | --- | --- | --- | --- | --- | --- | --- | --- | --- | --- | --- | --- | --- | --- | --- | --- | --- | --- | --- | --- | --- | --- | --- | --- | --- | --- | --- | --- | --- | --- | --- | --- | --- | --- | --- | --- | --- | --- | --- | --- |
